# The ubiquitin E3 ligase TRIM21 suppresses type I interferon signaling via STING degradation and ameliorates systemic autoimmunity

**DOI:** 10.1038/s12276-025-01490-5

**Published:** 2025-07-03

**Authors:** Da Som Kim, Youngjae Park, George C. Tsokos, Mi-La Cho, Seung-Ki Kwok

**Affiliations:** 1 The Rheumatism Research Center, Catholic Research Institute of Medical Science, College of Medicine, The Catholic University of Korea, Seoul, Republic of Korea; 2 Department of Medical Sciences, Graduate School of, The Catholic University of Korea, Seoul, Republic of Korea; 3 Division of Rheumatology, Department of Internal Medicine, Seoul St. Mary’s Hospital, College of Medicine, The Catholic University of Korea, Seoul, Republic of Korea; 4 Department of Medicine, Beth Israel Deaconess Medical Center, Harvard Medical School, Boston, MA USA; 5 Department of Medical Life Sciences, College of Medicine, The Catholic University of Korea, Seoul, Republic of Korea

**Keywords:** Autoimmunity, Immunology

## Abstract

Tripartite motif-containing 21 (TRIM21) is a cytoplasmic protein with E3 ubiquitin ligase activity. Although autoantibodies against TRIM21 are frequently detected in patients with systemic lupus erythematosus (SLE), its role in disease pathogenesis remains unclear. Here we demonstrate that TRIM21 directly interacts with the stimulator of interferon genes (STING) to regulate type I interferon (IFN) production. In both induced and spontaneous murine models of lupus, TRIM21 deficiency exacerbated lupus-like pathology and heightened IFN production after STING activation. By contrast, TRIM21 overexpression attenuated autoimmunity in lupus-prone mice. Mechanistically, TRIM21 binds to STING and promotes its degradation via the ubiquitin–proteasome pathway. In patients with SLE, TRIM21 expression levels inversely correlated with STING expression, type I IFN levels and autoantibody titers. These findings suggest that targeting the TRIM21–STING axis may offer a therapeutic strategy to reduce type I IFN production in SLE.

## TRIM21 modulates STING activity in systemic lupus erythematosus

Systemic lupus erythematosus (SLE) is an autoimmune disease in which the immune system attacks the body, causing inflammation and damage to various organs. Researchers have found that a protein called TRIM21 might play a role in controlling this disease. TRIM21 helps to regulate another protein, STING, which is involved in producing type I interferons (IFNs), molecules that can cause inflammation. They found that mice lacking TRIM21 had more severe lupus symptoms and higher levels of IFNs. By increasing TRIM21 levels in these mice, the symptoms improved. The researchers discovered that TRIM21 helps to break down STING, reducing IFN production and inflammation. The study suggests that low levels of TRIM21 in patients with SLE are linked to increased disease activity. This research highlights the potential of targeting the TRIM21–STING pathway as a new treatment approach for SLE.

This summary was initially drafted using artificial intelligence, then revised and fact-checked by the author.

## Introduction

Systemic lupus erythematosus (SLE) is a prototypic autoimmune disease causing inflammation and damage of multiple organs^1^. The disease is characterized by the production of autoantibodies and the formation of immune complexes in response to complex network interactions between immune cells of the innate and adaptive immune system^2^. The activation of type I interferons (IFNs) and subsequent increased expression of IFN-stimulated genes are frequently observed in patients with SLE^3^. Targeting the IFN activation pathway in patients with SLE has demonstrated clinical efficacy^4–6^. Yet, a significant proportion of patients, even among those with elevated IFN signatures, do not respond to such treatments, highlighting the need for a better understanding of the regulatory mechanisms involved in this pathway.

Several pathways are known to trigger the overproduction of IFN in SLE. One of the best-characterized mechanisms is mediated by the toll-like receptors (TLRs)^7–9^. TLR7 or TLR9 can recognize endosomal nucleic acids as well as autoantigen-derived immune complexes from extracellular sources and initiate intracellular pathways, including myeloid differentiation primary response 88 (MyD88) and IFN regulatory factors (IRFs), leading to the production of IFN^10^. It has been established that overexpression of TLR7 promotes lupus-like pathology, whereas its deletion reduces it^11,12^. However, more recently, a ‘non-TLR’-mediated pathway has been recognized that involves the recognition of cytoplasmic nucleic acids by a specific enzyme called cyclic GMP–AMP synthase (cGAS)^13^. This enzyme activates stimulator of IFN genes (STING), which is one of several cytoplasmic adaptor proteins^13^. Subsequently, STING enhances type I IFN production through the activation of other downstream proteins such as TANK-binding kinase 1 (TBK1) and IRF3^13^. Although the STING pathway can contribute to the pathogenesis of SLE by differentiating plasmacytoid dendritic cells (pDCs)^14^, the mechanisms that control its expression remain unknown.

Tripartite motif-containing 21 (TRIM21), also known as Ro52, is an essential cytoplasmic protein with E3 ubiquitin ligase activity^15^. Although autoantibodies targeting TRIM21 are frequently present in the sera of patients with various autoimmune diseases, including SLE^16^, the actual role of TRIM21 in the pathogenesis of SLE has not been elucidated. TRIM21-deficient mice develop lupus-like pathology by expanding the pool of T helper 17 (Th17) cells^17^ and enhancing the production of anti-double-stranded DNA (dsDNA) antibodies^18^. Several studies have suggested an interaction between TRIM21 and cytosolic nucleic acid sensors related to the STING pathway, such as DEAD-box helicase 41 (DDX41)^19^ and IFN gamma inducible protein 16 (IFI16)^20^. In view of these findings, we hypothesized that TRIM21 could act as a regulator of STING pathway-mediated IFN production in SLE.

Here, we report that TRIM21 ubiquitinates and degrades STING, thereby limiting the production of type I IFN and induced or spontaneous lupus-related pathology in mice. Our findings that the low levels of TRM21 in patients with SLE are associated with increased markers of disease activity are of translational value.

## Materials and methods

### Mice

C57BL/6 (B6) mice were purchased from Orient Bio. MRL/MpJ-*Fas*^*lpr*^ (MRL/*lpr*) mice were purchased from SLC. B6-*Trim21*^*tm1Hm*^ (*Trim21*^−/−^) mice (JAX stock no. 010724, Central Lab. Animal), B6.MRL-*Fas*^*lpr*^ (B6.*lpr*) mice (JAX stock no. 000482, Saeronbio) and *Trim21*^−/−^ X B6.*lpr* (*Trim21*^−/−^ B6.*lpr*) mice were bred in the animal facility at The Catholic University of Korea. Mice were maintained in groups of five in polycarbonate cages in a specific-pathogen-free environment. Mice were fed a standard mouse chow (1314, Altromin Spezialfutter GmbH & Co. KG) and housed under a 12-h light/12-h dark cycle, with a temperature of 20–26 °C and 50 ± 10% humidity. The Institutional Animal Care and Use Committee and the Department of Laboratory Animals at The Catholic University of Korea, Songeui Campus, were accredited by the Korea Excellence Animal Laboratory Facility from Korea Food and Drug Administration in 2017 and reaccredited in 2021. Association for Assessment and Accreditation of Laboratory Animal Care (AAALAC). International full accreditation was also acquired in 2018 and reaccredited in 2022. All animal research procedures were provided in accordance with the Laboratory Animals Welfare Act, the Guide for the Care and Use of Laboratory Animals and the Guidelines and Policies for Rodent Experiment provided by the Institutional Animal Care and Use Committee of the College of Medicine, The Catholic University of Korea (approval nos. CUMS-2020-0112-02, CUMS-2021-0256-01, CUMS-2022-0174-01 and CUMS-2023-0184-01).

### Plasmid vector construction

Mouse Trim21 (clone ID: mMU001736) and human TRIM21 (clone ID: hMU000921) cDNA fragments (Korea Human GeneBank, Medical Genomics Research Center, KRIBB, Daejeon, Korea) were cloned into the pCMV-SPORT6 vector. Sequencing of *Escherichia coli* strain DH5α containing the mouse *Trim21* or human *TRIM21* overexpression vector were performed at Cosmo Genetech, and these vectors were incubated in Luria Bertani (LB) broth (110285, Millipore) at 37 °C and 150 rpm for 16 h. The cells were collected by centrifugation, and the vector was purified using the NucleoBond Xtra Maxi EF Kit (740424.50, MACHEREY-NAGEL). The pmCherry-C1 mock vector was purchased from Takara Bio. pmCherry-C1-*mTrim21* (Addgene plasmid no. 105516; http://n2t.net/addgene:105516; RRID: Addgene_105516), pmCherry-C1-*mTrim21ΔRING-Box* (Addgene plasmid no. 105517; http://n2t.net/addgene:105517; RRID: Addgene_105517) and pmCherry-C1-*mTrim21ΔPRYSPRY* (Addgene plasmid no. 105518; http://n2t.net/addgene:105518; RRID: Addgene_105518) were gifts from Melina Schuh^21^.

### In vivo gene delivery

Eight-week-old female MRL/*lpr* mice were injected intravenously with 200 μg mock or mouse *Trim21* overexpression vector in 1 ml of saline once a week for 8 weeks. At the end of the experiment, the mice were euthanized and perfused with phosphate-buffered saline and 10% formalin solution (HT501320, Sigma-Aldrich).

### Resiquimod (R848) in vivo

Eight-week-old female B6 and *Trim21*^−/−^ mice were treated via epicutaneous application of 50 μg of R848 (SML0196, Sigma-Aldrich) dissolved in 10 μl of acetone, or acetone alone as a vehicle, to one ear, three times a week for 31 days.

### PAS staining and scoring

Kidney tissues were fixed in 4% paraformaldehyde, embedded in paraffin, cut into 4-μm sections and stained with a periodic acid Schiff (PAS) stain kit (ab150680, Abcam). Kidney histological pathology was evaluated using the lupus nephritis classification system, as described in ref. ^22^.

### Confocal microscopy

Murine spleen tissue was cryosectioned into 5-μm-thick slices. Human peripheral blood mononuclear cells (PBMCs) were placed in the appropriate well of a cytospin chamber (Thermo Fisher Scientific) and centrifuged at 700 rpm for 3 min. Murine spleen tissue cryosections or human PBMCs were fixed with methanol–acetone and stained. The following antibodies were used. Anti-mouse CD19 (60112) was from STEMCELL Technologies. Alexa Fluor 488 anti-rat immunoglobulin (Ig)G secondary antibody (A-11006), Alexa Fluor 488 anti-rabbit IgG secondary antibody (A-11034), APC anti-mouse IgG secondary antibody (A-865) and FITC anti-sheep IgG secondary antibody (A16049) were from Invitrogen (Thermo Fisher Scientific). Anti-CXCL10 (MBS8502352) was from MyBioSource. PE anti-rabbit IgG secondary antibody (4050-09) was from SouthernBiotech. PE anti-mouse PDCA1 (127010) was from BioLegend. Anti-IFNα (ab193055) was from Abcam. Anti-STING (NBP2-24683) was from Novus Biologicals (Bio-Techne). Anti-IFNα (MAB93454) and anti-TRIM21 (AF6219) were from R&D Systems (Bio-Techne). 4′,6-Diamidino-2-phenylindole (DAPI; D1306, Invitrogen) was used for nuclear staining. The stained samples were visualized by confocal microscopy (LSM 700; Carl Zeiss).

### Isolation of murine splenocytes

Mouse spleens were ground using sterilized glass slides with frosted ends, and red blood cells were lysed in ACK buffer (A1049201, Gibco, Thermo Fisher Scientific). The remaining splenocytes were filtered through a 40-μm cell strainer (352340, Falcon) and maintained in RPMI 1640 medium (Gibco) containing 5% fetal bovine serum (FBS; 16000044, Gibco).

### Isolation of spleen B cells and stimulation

To purify splenic B cells, the splenocytes were incubated with CD19- (130-121-301) or B220- (130-049-501) coated magnetic beads and isolated on Magnetic-Activated Cell Sorting (MACS) separation columns (Miltenyi Biotec). B220^+^ B cells were stimulated with CD40 ligand (1 μg/ml; 315-15, PeproTech, Thermo Fisher Scientific), anti-IgM (10 μg/ml; 115-006-020, Jackson ImmunoResearch Laboratories) and IL-4 (20 ng/ml; 404-ML, R&D Systems).

### siRNA transfection

Small interfering RNA (siRNA) for *Trim21* (4392422, siRNA ID: s13462) and negative control (4390843) were acquired from Ambion (Invitrogen). Spleen CD19^+^ B cells from 8-week-old female MRL/*lpr* mice were transfected using Accell siRNA Delivery Media (B-005000, Dharmacon) and 1 μM siRNA. One day later, the cells were stimulated with CD40 Ligand (100 ng/ml; 315-15, PeproTech) and IL-4 (10 ng/ml; 404-ML, R&D Systems) for 3 days.

### Cell culture and vector transfection

HEK293 and NIH3T3 cells were cultured with in DMEM (Gibco) medium containing 10% FBS for 9 h. The cells were stimulated with MG132 (C2211, Sigma-Aldrich) or NH_4_Cl (A9434, Sigma-Aldrich) for 15 h. Mock and *TRIM21* overexpression vectors were transfected with X-tremeGENE HP DNA Transfection Reagent (Roche).

### Western blot analysis

Cells were lysed in RIPA buffer containing protease and phosphatase inhibitor cocktail (both from Thermo Fisher Scientific). Lysates were centrifuged at 4 °C and 14,000 rpm for 15 min. Protein concentration was determined using BCA protein assay kits (Thermo Fisher Scientific). Proteins were separated by sodium dodecyl sulfate–polyacrylamide gel electrophoresis (SDS–PAGE) and transferred to membranes (Cytiva). Membranes were then probed with antibodies against TRIM21 (ab207728, Abcam), STING (13647, Cell Signaling Technology), IFI16 (ab185812, Abcam), DDX41 (15076, Cell Signaling Technology), GAPDH (ab181602, Abcam) and β-actin (sc-47778, Santa Cruz Biotechnology). After washing, HRP-conjugated secondary antibodies were added and incubated. Hybridized bands were detected by enhanced chemiluminescence (Thermo Fisher Scientific) on X-ray film (AGFA HealthCare). Protein detection was performed using a manual western blotting system and the SNAP i.d. 2.0 Protein Detection System (Millipore). Quantitation of western blots was performed using ImageJ.

### IP and western blotting

Immunoprecipitation (IP) was performed by incubating Dynabeads Protein G (10003D, Invitrogen) under rotation with antibodies against STING (13647, Cell Signaling Technology) or normal rabbit IgG (sc-2027, Santa Cruz Biotechnology) at 4 °C for 1 h. Cell lysates were added and further incubated at 4 °C for 2 h; then bound complexes were eluted. Western blotting was performed using antibodies against STING (13647, Cell Signaling Technology), TRIM21 (ab207728, Abcam), ubiquitin (sc-8017, Santa Cruz Biotechnology), mCherry (ab183628, Abcam) and GAPDH (ab181602, Abcam).

### In vitro ubiquitination assay

An in vitro ubiquitination assay was performed using TRIM21/RO52 recombinant protein (MBS1576070, MyBioSource), STING recombinant protein (MBS8305104, MyBioSource) and E3 Ligase Auto-Ubiquitylation Assay Kit (ab139469, Abcam). The reaction mixture was incubated at 37 °C for 1 h. Proteins were separated by SDS–PAGE and transferred to membranes (Cytiva). Membranes were then probed with antibodies against Ubiquitin (sc-8017, Santa Cruz Biotechnology), TRIM21 (ab207728, Abcam) and STING (13647, Cell Signaling Technology).

### Flow cytometry analysis

For intracellular cytokine staining, cells were stimulated with 25 ng/ml phorbol 12-myristate 13-acetate (P8139, Sigma-Aldrich) and 250 ng/ml ionomycin (I0634, Sigma-Aldrich) with the addition of GolgiStop (554724, BD Biosciences) for 4 h. For surface marker staining, single-cell suspensions were incubated with fluorochrome-labeled antibodies at 4 °C for 30 min. After surface staining, cells were fixed and permeabilized with Cytofix/Cytoperm (554715, BD Biosciences) or Foxp3/Transcription Factor Staining Buffer kit (00-5523-00, Invitrogen) according to the manufacturer’s instructions. After washing with Perm/Wash buffer, antibodies for intracellular staining were added at 4 °C for 30 min. The following antibodies were used. Anti-mouse CD16/CD32 (Fc Block; 553142), APC anti-mouse CD11c (550261), BV421 anti-mouse Siglec-H (566581), PE-Cy7 anti-mouse CD19 (552854), PE anti-mouse CD138 (553714), FITC anti-mouse T and B cell activation antigen (GL-7; 553666), PE anti-mouse IL-4 (554435), anti-human Fc block (564220), PE-Cy5 anti-human CD19 (555414), BV510 anti-human CD11c (563026) and PE-Cy7 anti-human CD123 (560826) were from BD Biosciences. PerCP-Cyanine5.5 anti-mouse CD19 (45-0193-82) was from Invitrogen. eFluor 780 Fixable Viability Dye (65-0865-14), Alexa Fluor 488 anti-rabbit IgG secondary antibody (A-11008), PE anti-mouse CD1d (12-0011-83), PE anti-mouse CD5 (12-0051-82), APC anti-mouse IL-10 (17-7101-82), PerCP-Cyanine5.5 anti-mouse IL-17A (45-7177-82), PE anti-mouse/human B220 (12-0452-83), PerCP-Cyanine5.5 anti-mouse CD4 (45-0042-82), FITC anti-mouse IL-17A (11-7177-81) and PE anti-mouse Foxp3 (12-5773-82) were from eBioscience (Invitrogen). PE anti-mouse PDCA1 (127010), Pacific Blue anti-mouse CD90.2 (105324), PE anti-mouse CD8a (100708), APC anti-mouse IFNγ (505810), APC anti-mouse CD25 (102012), APC anti-human CD303 (354206) and PE anti-human CD304 (354504) were from BioLegend. Alexa Fluor 488 rabbit IgG isotype control antibody (4340), anti-STING antibody (13647), anti-TBK1 antibody (38066), anti-phospho-TBK1 antibody (5483) and Alexa Fluor 488 anti-phospho-IRF3 antibody (53539) were from Cell Signaling Technology. FITC anti-mouse IFNα (22100-3) was from PBL Assay Science. Anti-phospho-STING antibody (PA5-105674) and anti-IRF3 antibody (MA5-32348) were from Invitrogen. Anti-CXCL10 antibody (MBS8502352) was from MyBioSource. Anti-TRIM21 antibody (ab207728) was from Abcam. Data were obtained on an LSRFortessa (BD Biosciences) and analyzed with FlowJo software (Tree Star).

### Quantitative polymerase chain reaction (qPCR)

Total RNA was extracted using TRI Reagent (TR 118, Molecular Research Center, OH, USA), and cDNA was synthesized with a Dyne first-stranded cDNA synthesis kit (BN615, Dyne Bio) according to the manufacturer’s protocol. PCR amplification was performed with an StepOnePlus Real-Time PCR System (Applied Biosystems, Thermo Fisher Scientific) and SensiFAST SYBR Hi-ROX (Meridian Bioscience) according to the manufacturer’s instructions. The following primers were used for mouse samples: *Blimp1*, 5′-CTGTCAGAACGGGATGAACA-3′ (sense), 5′-TGGGGACACTCTTTGGGTAG-3′ (antisense); *Xbp1*, 5′-AAGAAAGCCCGGATGAGC-3′ (sense), 5′-AGCGTGTTCTTAACTCCTGG-3′ (antisense); *Bcl6*, 5′-CCCTGTGAA ATCTGTGGCACTC-3′ (sense), 5′-ACACGCGGTATTGCACCTTG-3′ (antisense); *Pax5*, 5′-CACAGTCCTACCCTATTGTCAC-3′ (sense), 5′-TCCAGAAAATTCACTCCCAGG-3′ (antisense); *Trim21*, 5′-CCCAGACCTAACAAGCACATG-3′ (sense), 5′-TCTCCCATCCTCACTTGTCTC-3′ (antisense); *Sting1*, 5′-TATACCTCAGTTGGATGTTTGGC-3′ (sense), 5′-CTGGAGTCAAGCTCTGAAGGC-3′ (antisense); and *Actb*, 5′-GAAATCGTGCGTGACATCAAAG-3′ (sense), 5′-TGTAGTTTCATGGATGCCACAG-3′ (antisense). The levels of messenger RNA (mRNA) expression were normalized relative to that of *Actb* mRNA. The following primers were used for human samples: *TRIM21*, 5′-AAGCTCCAGGTGGCATTAG-3′ (sense), 5′-ACAAACTCTGCGTGAATCCTAG-3′ (antisense); *CXCL10*, 5′-CCTTATCTTTCTGACTCTAAGTGGC-3′ (sense), 5′-ACGTGGACAAAATTGGCTTG-3′ (antisense); *IFNA2*, 5′-AGAAATACAGCCCTTGTGCC-3′ (sense), 5′-GAGCTGGCATACGAATCAATG-3′ (antisense); and *ACTB*, 5′-CATGTACGTTGCTATCCAGGC-3′ (sense), 5′-CTCCTTAATGTCACGCACGAT-3′ (antisense). The levels of mRNA expression were normalized relative to that of *ACTB* mRNA.

### Measurement of IgG

Murine blood was collected from the orbital sinus, and serum samples were stored at −20 °C until use. Serum levels of dsDNA antibodies were measured using poly-l-lysine solution (P4707, Sigma-Aldrich), dsDNA-cellulose powder (D8515, Sigma-Aldrich) and mouse IgG detection antibody (Bethyl Laboratories). Murine serum and cultured supernatant levels of IgG were measured using anti-mouse IgG antibody (Bethyl Laboratories). Absorbances were determined using an enzyme-linked immunosorbent assay (ELISA) microplate reader (Molecular Devices).

### Urine albumin and creatinine assays

Spot urine samples were collected using aseptic microtubes. Urine albumin and creatinine concentrations were measured using anti-mouse albumin antibody (Bethyl Laboratories) and creatinine assay kit (KGE005, R&D Systems).

### Isolation of human PBMCs

Human blood was separated from buffy coats using Ficoll-Paque Plus (Cytiva). Red blood cells were removed, and the PBMCs were maintained in RPMI 1640 medium containing 10% FBS. All procedures were approved by the ethics committee of Seoul St. Mary’s Hospital (approval no. KC18TESI0519), and all participating patients provided their informed consent.

### Proximity ligation assay

The interaction between TRIM21 and STING was validated using antibodies against TRIM21 (15-060, ProSci) and STING (sc-518172, Santa Cruz Biotechnology) in human PBMCs and mouse splenocytes. The interaction between mCherry-tagged TRIM21 and STING was confirmed using antibodies against mCherry (ab183628, Abcam) and STING in NIH3T3 cells overexpressing pmCherry-C1-*mTrim21*, pmCherry-C1-*mTrim21ΔRING-Box* and pmCherry-C1-*mTrim21ΔPRYSPRY*. Proximity ligation assay (PLA) was performed using the NaveniFlex Cell MR Atto647N (NC.MR.100 Atto647N, Navinci Diagnostics) according to the manufacturer’s instructions. The stained samples were visualized by confocal microscopy (LSM 700).

### Statistical analysis

Statistical analyses were performed using GraphPad Prism (version 8 for Windows; GraphPad Software). *P* values were calculated using the two-tailed paired *t*-test, one-way or two-way analysis of variance (ANOVA) and Pearson’s correlation analysis. *P* < 0.05 was considered statistically significant. *P* values are presented within each figure and figure legend.

## Results

### TRIM21 gene therapy attenuates autoimmune phenotypes in lupus-prone mice

**Fig. 1:**
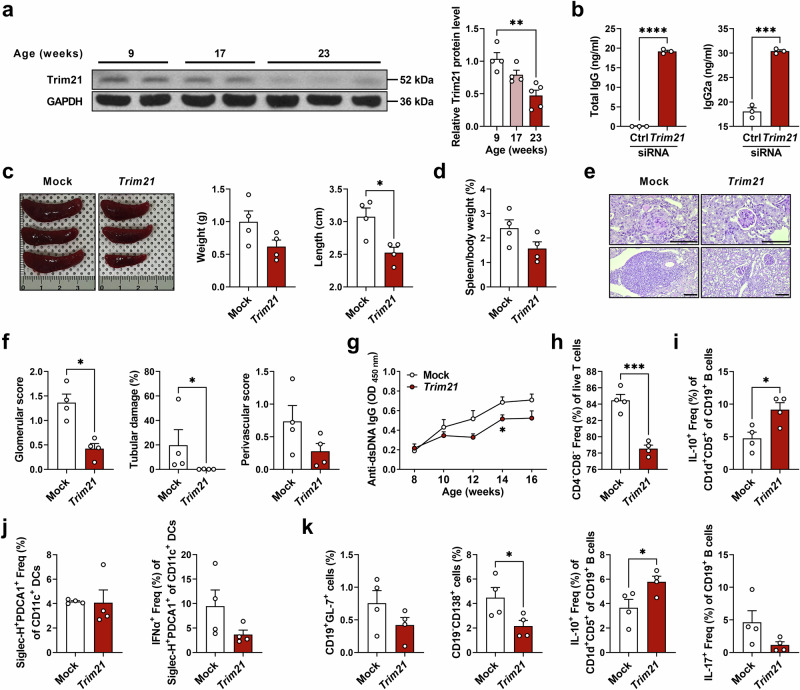
TRIM21 gene therapy attenuates autoimmune phenotypes in lupus-prone mice. **a** Western blots and densitometry analysis of Trim21 in splenocytes from female MRL/*lpr* mice (9 weeks, *n* = 4; 17 weeks, *n* = 4; 23 weeks, *n* = 5). **b** Spleen CD19^+^ B cells from 8-week-old female MRL/*lpr* mice were transfected with *Trim21* siRNA and stimulated with CD40 ligand and IL-4 for 4 days. Levels of total IgG and IgG2a in the culture supernatant were determined using ELISA. **c**–**k** Eight-week-old female MRL/*lpr* mice were injected with mock (*n* = 4) or *Trim21* overexpression (*n* = 4) vector for 8 weeks: weights and lengths of spleens (**c**) and percentage of spleen weight relative to body weight (**d**); representative photomicrographs of PAS-stained kidney tissues of MRL/*lpr* mice (**e**) with histopathologic analysis (**f**) (in **e**, glomerular and tubular images (top) and vascular images (bottom) are shown; scale bar, 100 μm); serum levels of anti-dsDNA antibodies (total IgG) in MRL/*lpr* mice measured using ELISA (**g**); flow cytometric analysis of double-negative T cells in peripheral blood of 16-week-old MRL/*lpr* mice (live T cells were defined as Fixable Viability Dye^−^CD90.2^+^ cells) (**h**); flow cytometric analysis of regulatory B cells (**i**) and pDCs or IFNα-producing pDCs (**j**) in splenocytes of 16-week-old MRL/*lpr* mice; flow cytometric analysis of GC B cells, plasma B cells and IL-10- or IL-17-producing B cells in peripheral blood of 16-week-old MRL/*lpr* mice (**k**). All data are shown as mean ± s.e.m. Statistical analyses were performed using one-way ANOVA (**a**), two-tailed paired *t*-test (**b**–**d**, **f** and **h**–**k**) or two-way ANOVA (**g**). *P < 0.05, **P < 0.01, ***P < 0.001, ****P < 0.0001.

MRL/*lpr* mice are widely used to study aspects of SLE pathogenesis^23^. Previous studies have reported that TRIM21-deficient MRL/*lpr* mice presented with exacerbated lupus-like pathology, including increased levels of serum dsDNA antibody and urine protein, compared with wild-type (WT) MRL/*lpr* mice^18^. First, we determined the levels of expression of TRIM21 in MRL/*lpr* mice and found that they decrease gradually as mice aged (Fig. 1a). Compared with younger mice, B cells (Supplementary Fig. 1a–c) and pDCs (Supplementary Fig. 1d–f) from the older mice had a highly activated STING pathway, while TRIM21 expression was decreased (Supplementary Fig. 1a–f). When TRIM21 expression was inhibited in B cells of 8-week-old MRL/*lpr* mice using siRNA, IgG production significantly increased (Fig. 1b). Considering the decreased TRIM21 expression in lupus-prone mice, we wished to determine whether TRIM21 overexpression by in vivo gene therapy could ameliorate lupus-like phenotypes in MRL/*lpr* mice. MRL/*lpr* mice injected with a *Trim21* overexpression vector displayed reduced autoimmune features, including splenomegaly (Fig. 1c, d), renal inflammation (Fig. 1e, f), circulating anti-dsDNA production (Fig. 1g) and double-negative T cells in the peripheral blood (Fig. 1h). In addition, flow cytometric analyses of immune cells in spleens (Fig. 1i, j and Supplementary Fig. 1g) and peripheral blood (Fig. 1k) showed that the IL-10-expressing B (regarded as regulatory B; Fig. 1i) and regulatory T (T_reg_) cells were significantly increased, while Th17 cells were decreased (Supplementary Fig. 1g) compared with control mice. Spleen IFNα-expressing pDCs (Fig. 1j), peripheral blood germinal center (GC) B cells, plasma B cells and IL-17-expressing B cells were decreased, whereas regulatory B cells were increased in TRIM21 overexpressed MRL/*lpr* mice (Fig. 1k). Collectively, TRIM21 overexpression by in vivo gene therapy attenuated lupus-like phenotypes and exerted anti-inflammatory effects on immune cell profiles in MRL/*lpr* mice.

### IFNα-producing pDCs are increased in TRIM21-deficient mice

**Fig. 2:**
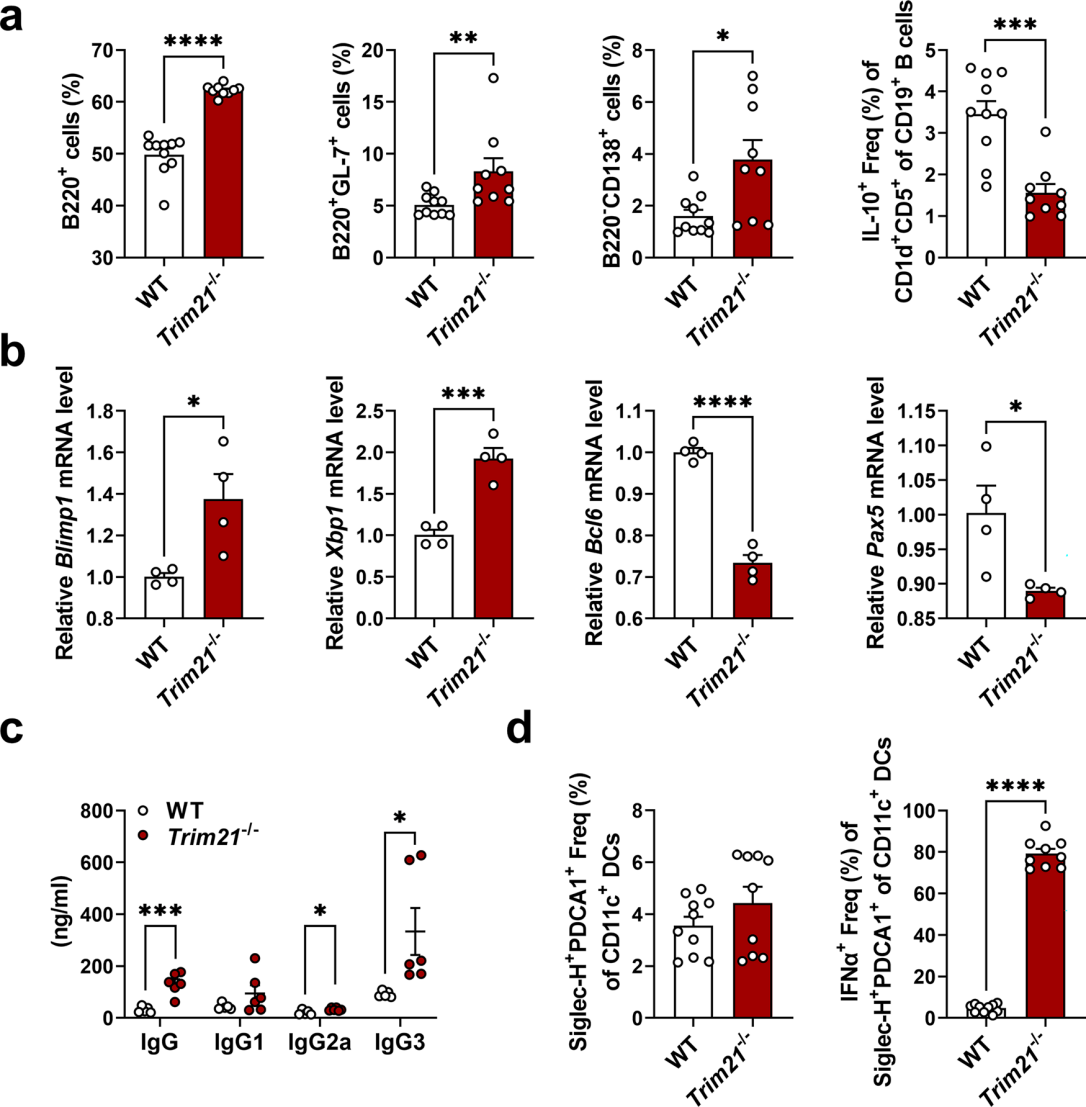
IFNα-producing pDCs are increased in TRIM21-deficient mice. **a**, **d** Flow cytometric analysis of B cells (**a**) and pDCs (**d**) in splenocytes from WT B6 (*n* = 10) and *Trim21*^−/−^ (*n* = 9) mice. In **a**, total B220^+^ cells, GC B cells, plasma B cells and regulatory B cells were analyzed. In **d**, pDCs and IFNα-producing pDCs were analyzed. **b**, **c** Splenic B220^+^ B cells were stimulated with CD40 ligand, anti-IgM and IL-4. mRNA levels of *Blimp1*, *Xbp1*, *Bcl6* and *Pax5* in the cells were determined using qPCR (**b**), and levels of total IgG, IgG1, IgG2a and IgG3 in the culture supernatant were determined using ELISA (**c**). All data are shown as mean ± s.e.m. Statistical analyses were performed using two-tailed paired *t*-test (**a**, **b** and **d**) or two-way ANOVA (**c**). *P < 0.05, **P < 0.01, ***P < 0.001, ****P < 0.0001.

In view of the immune-modulatory effects of TRIM21 in MRL/*lpr* mice, we investigated its effect on immune cells, including pDCs, using TRIM21-deficient (*Trim21*^−/−^) mice. In *Trim21*^−/−^ mice, populations of total B cells and the differentiation to GC B cells and plasma B cells were significantly increased both ex vivo and under in vitro stimulation (Fig. 2a and Supplementary Fig. 2a). Meanwhile, the proportion of regulatory B cells was significantly reduced in *Trim21*^−/−^ mice (Fig. 2a). Furthermore, TRIM21 deficiency resulted in significantly increased expression of *Blimp1* and *Xbp1* and reduced *Bcl6* and *Pax5* in splenic B cells (Fig. 2b), subsequently leading to increased differentiation of plasma B cells and production of total IgG, IgG2a and IgG3 (Fig. 2c). Interestingly, lack of TRIM21 did not change the percentages of pDCs, but it resulted in remarkably increased percentages of IFNα-producing pDCs (Fig. 2d). Based on these findings, the differentiation to inflammatory B cells, including GC B cells and plasma B cells, and the induction of IFNα-producing pDCs are increased when TRIM21 is absent.

### TRIM21 deficiency exacerbates lupus pathology by activating the STING pathway in two different murine models

**Fig. 3:**
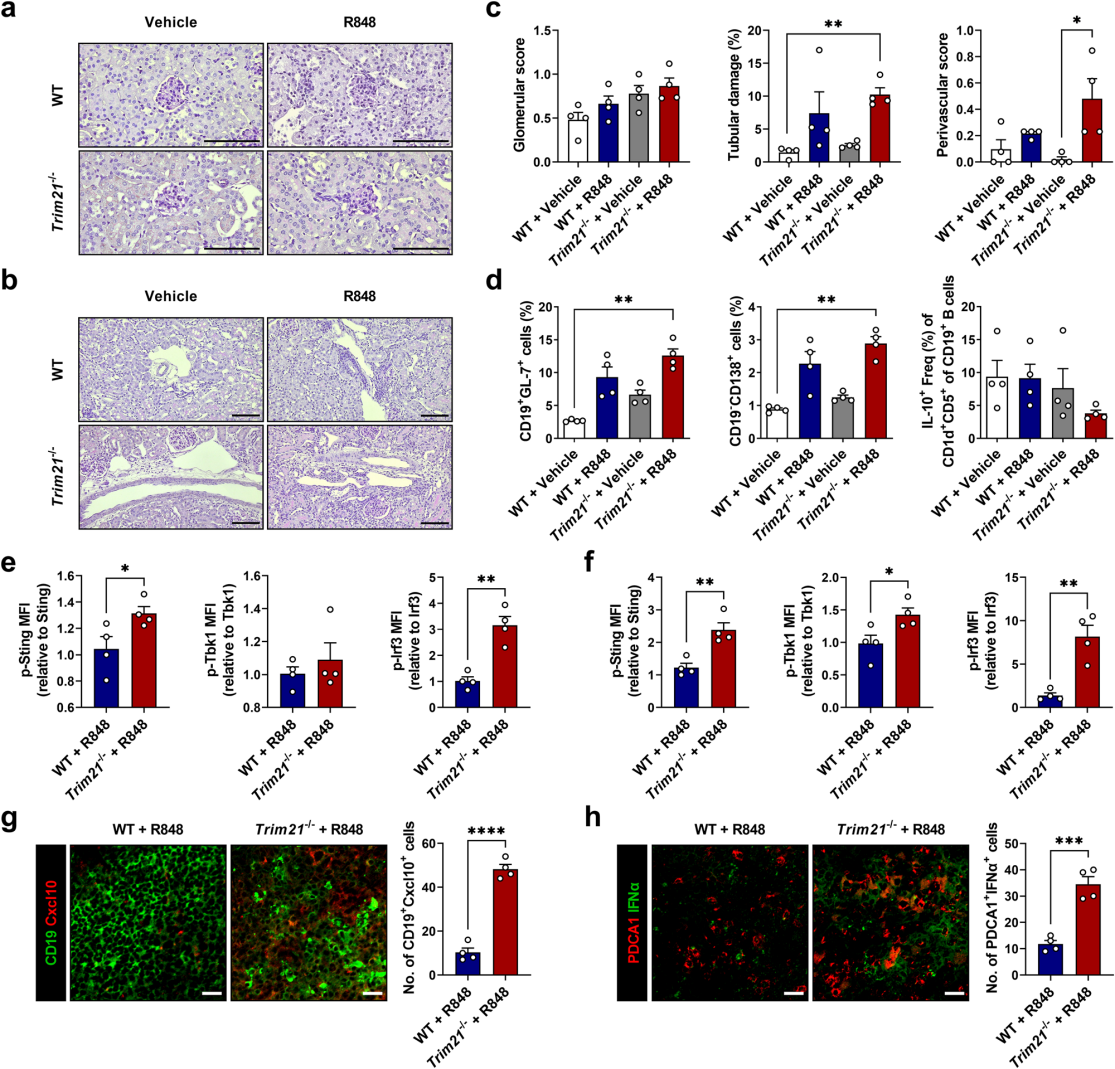
TRIM21 deficiency exacerbates lupus manifestations through STING pathway activation in R848-treated mice. Eight-week-old female WT B6 and *Trim21*^−/−^ mice were treated with vehicle (acetone alone, *n* = 4) or R848 (*n* = 4) for 31 days. **a**–**c** Representative photomicrographs of PAS-stained kidney tissues of mice with histopathologic analyses. Glomerular and tubular images (**a**) and vascular images (**b**) are shown (scale bar, 100 μm). **d** Flow cytometric analysis of GC B cells, plasma B cells and regulatory B cells in splenocytes from mice. **e**, **f** Flow cytometric analysis of the STING pathway in splenocytes from mice. The bar graphs represent the relative Mean Fluorescence Intensity (MFI) values analyzed in CD19^+^ B cells (**e**) or Siglec-H^+^PDCA1^+^ Freq (%) of CD11c^+^ DCs (**f**). **g**, **h** Confocal images of spleen sections stained for CD19 (green) and Cxcl10 (red) (**g**) or PDCA1 (red) and IFNα (green) (**h**) (scale bar, 20 μm). Cell counts are shown on the right. All data are shown as mean ± s.e.m. Statistical analyses were performed using one-way ANOVA (**c** and **d**) or two-tailed paired *t*-test (**e**–**h**). *P < 0.05, **P < 0.01, ***P < 0.001, ****P < 0.0001.

The results described above indicate that TRIM21 deficiency induces an enhanced inflammatory response with increased numbers of active B cells and IFNα-producing pDCs. Therefore, we evaluated the effect of TRIM21 deficiency on lupus manifestations in two independent lupus murine models. In the first, we induced lupus in WT B6 and *Trim21*^−/−^ mice using R848, a TLR7 agonist^24^. R848-treated *Trim21*^−/−^ mice had a lower body weight (Supplementary Fig. 2b) and increased kidney inflammation compared with WT mice (Fig. 3a–c). Also, R848-treated TRIM21-deficient mice had increased proportions of GC B cells and plasma B cells while regulatory B cells were diminished (Fig. 3d). The proportions of Th1, Th2, Th17 cells and IFNα-producing pDCs were significantly increased whereas those of T_reg_ cells were decreased in *Trim21*^−/−^ mice compared with WT mice (Supplementary Fig. 2c, d). In addition, in vitro stimulation of TRIM21-deficient lymphocytes with R848 resulted in significantly higher IFNα production (Supplementary Fig. 2e). Next, to determine whether TRIM21 deficiency affects type I IFN production through the STING pathway in this model, we measured the expression levels of STING pathway-related molecules in both murine B cells and pDCs. In *Trim21*^−/−^ mice, phosphorylated Sting, Tbk1 and Irf3 were expressed at significantly higher levels in both cell subsets, indicating STING pathway activation (Fig. 3e, f). Furthermore, confocal imaging of murine spleen tissues revealed significantly higher numbers of Cxcl10-expressing B cells and IFNα-producing pDCs in TRIM21-deficient mice compared with WT mice (Fig. 3g, h), implying activated inflammatory features in both B cells and pDCs.

**Fig. 4:**
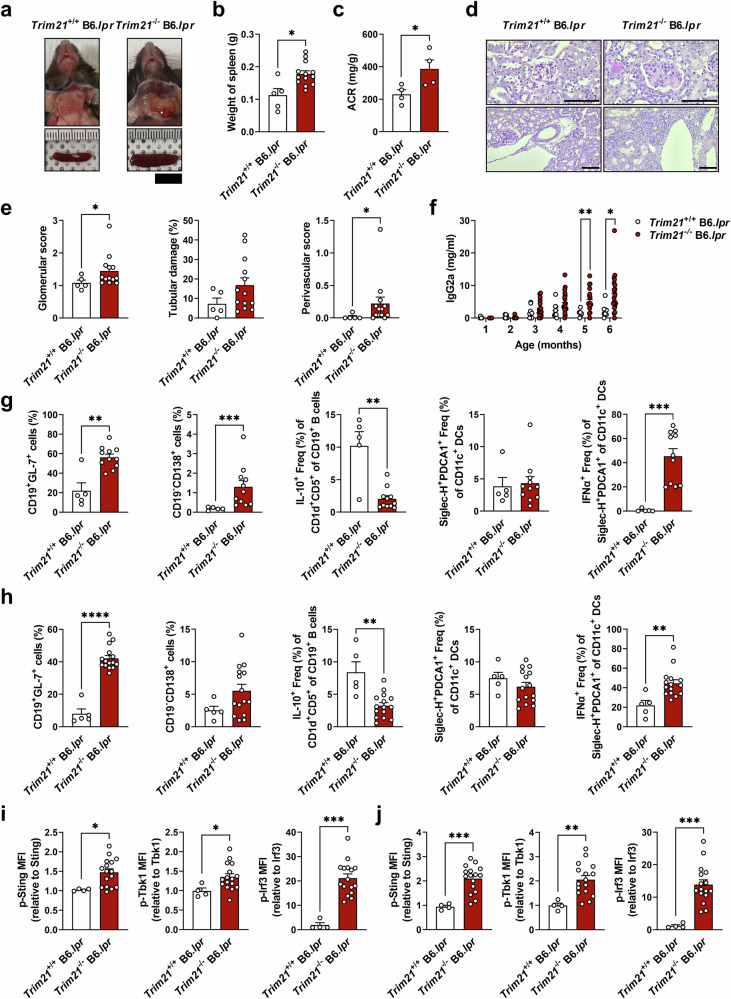
TRIM21 deficiency exacerbates lupus manifestations through STING pathway activation in B6.*lpr* mice. **a** Representative images of 7-month-old WT (*Trim21*^+/+^) and *Trim21*^−/−^ B6.*lpr* mice with their spleen (scale bar, 1 cm). **b** The spleen weights of 7-month-old *Trim21*^+/+^ (*n* = 5) and *Trim21*^−/−^ (*n* = 13) B6.*lpr* mice. **c** ACR measured from urine of 10-month-old *Trim21*^+/+^ (*n* = 4) and *Trim21*^−/−^ (*n* = 4) B6.*lpr* mice. **d**, **e** Representative photomicrographs of PAS-stained kidney tissues of 7-month-old *Trim21*^+/+^ (*n* = 5) and *Trim21*^−/−^ (*n* = 13) B6.*lpr* mice with histopathologic analyses. Glomerular and tubular image (top) and vascular image (bottom) are shown (**d**; scale bar, 100 μm). **f** IgG2a measured from serum of *Trim21*^+/+^ and *Trim21*^−/−^ B6.*lpr* mice using ELISA. **g** Flow cytometric analysis of GC B cells, plasma B cells, regulatory B cells, pDCs and IFNα-producing pDCs in peripheral blood of 5-month-old *Trim21*^+/+^ (*n* = 5) and *Trim21*^−/−^ (*n* = 11) B6.*lpr* mice. **h** Flow cytometric analysis of GC B cells, plasma B cells, regulatory B cells, pDCs and IFNα-producing pDCs in splenocytes of 7-month-old *Trim21*^+/+^ (*n* = 5) and *Trim21*^−/−^ (*n* = 15) B6.*lpr* mice. **i**, **j** Flow cytometric analysis of the STING pathway in splenocytes. The bar graphs represent the relative MFI values analyzed in CD19^+^ B cells (**i**) or Siglec-H^+^PDCA1^+^ Freq (%) of CD11c^+^ DCs (**j**). All data are shown as mean ± s.e.m. Statistical analyses were performed using two-tailed paired *t*-test (**b**, **c**, **e** and **g**–**j**) or two-way ANOVA (**f**). *P < 0.05, **P < 0.01, ***P < 0.001, ****P < 0.0001. ACR albumin/creatinine ratio.

To examine the effects of TRIM21 deficiency in a spontaneous lupus-prone mouse, we generated TRIM21-deficient B6.*lpr* (*Trim21*^−/−^ B6.*lpr*) mice. Compared with WT B6.*lpr* (*Trim21*^+/+^ B6.*lpr*) mice, *Trim21*^−/−^ B6.*lpr* mice showed more severe splenomegaly (Fig. 4a, b), lymph node enlargement (Supplementary Fig. 3a) and renal inflammation as well as albuminuria (Fig. 4c–e). Serum IgG2a levels were significantly higher in *Trim21*^−/−^ B6.*lpr* mice than in WT mice (Fig. 4f), and serum dsDNA antibody levels showed similar tendencies (Supplementary Fig. 3b). Flow cytometric analyses of immune cells in spleen and peripheral blood showed significantly elevated proportions of GC B cells, plasma B cells with higher Cxcl10 expression and IFNα-producing pDCs in *Trim21*^−/−^ B6.*lpr* mice, while regulatory B cells were reduced (Fig. 4g, h and Supplementary Fig. 3c). CD19^+^ B cells and pDCs from TRIM21-deficient B6.*lpr* mice showed higher expression of active Sting, Tbk1 and Irf3, suggesting enhanced STING pathway activation compared to WT mice (Fig. 4i, j). Collectively, in two different lupus murine models, TRIM21 deficiency induced more severe lupus-like phenotype with increased IFNα production and STING pathway activation.

### TRIM21 directly degrades STING via the ubiquitin–proteasome pathway

**Fig. 5:**
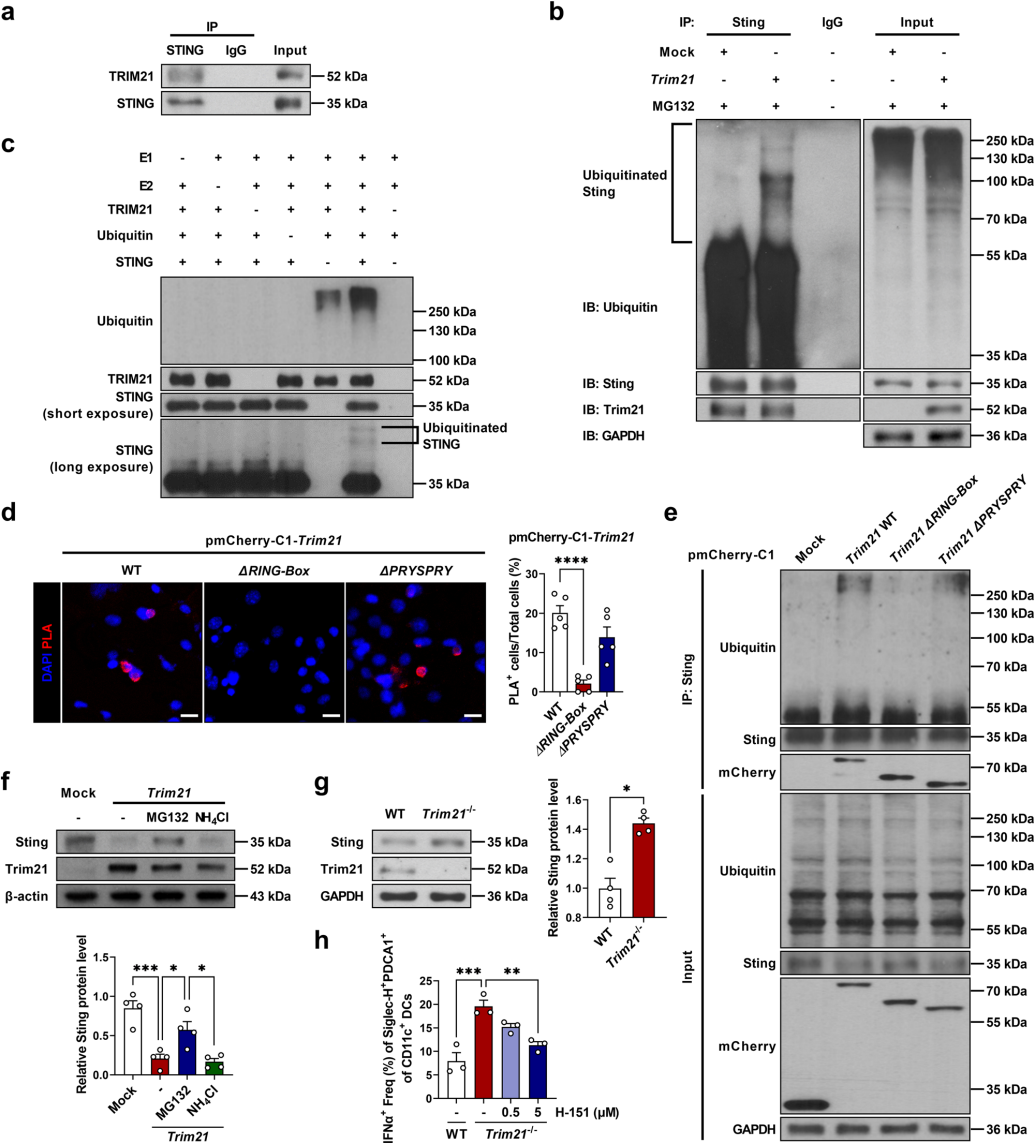
TRIM21 directly degrades STING via the ubiquitin–proteasome pathway. **a** HEK293 cells were immunoprecipitated with an anti-STING antibody or normal IgG, and immunoblotted using anti-TRIM21 and anti-STING antibodies. **b** NIH3T3 cells were transfected with mock or mouse *Trim21* overexpression vector (3 μg) with MG132 (5 μM) treatment. The cells were immunoprecipitated with anti-Sting antibodies or normal IgG and immunoblotted using anti-ubiquitin, anti-Sting and anti-Trim21 antibodies. **c** In vitro ubiquitination assay using recombinant TRIM21 and STING proteins. The reaction mixture was incubated at 37 °C for 1 h. Proteins were separated by SDS–PAGE and immunoblotted using anti-ubiquitin, anti-TRIM21 and anti-STING antibodies. **d** PLA to detect protein interactions between mCherry and Sting in NIH3T3 cells overexpressing pmCherry-C1-*Trim21*, pmCherry-C1-*Trim21ΔRING-Box* and pmCherry-C1-*Trim21ΔPRYSPRY*. Nuclei were stained with DAPI (scale bar, 20 μm). **e** NIH3T3 cells were transfected with pmCherry-C1 mock or *Trim21* overexpression vectors and treated with MG132 (5 μM). Cells were immunoprecipitated with anti-Sting antibodies and immunoblotted with anti-ubiquitin, anti-Sting and anti-mCherry antibodies. **f** Western blots and densitometry analysis of cells for Sting and Trim21. NIH3T3 cells were treated with MG132 (5 μM) or NH_4_Cl (10 mM). **g** Western blots and densitometry analysis of splenocytes from WT B6 and *Trim21*^−/−^ mice for Sting and Trim21. **h** Splenocytes from WT B6 and *Trim21*^−/−^ mice were stimulated with H-151 for 2 days. IFNα-producing pDCs in the cells were analyzed using flow cytometry. All data are shown as mean ± s.e.m. Statistical analyses were performed using one-way ANOVA (**d**, **f** and **h**) or two-tailed paired *t*-test (**g**). *P < 0.05, **P < 0.01, ***P < 0.001, ****P < 0.0001.

Considering that both in vivo and ex vivo results implied a relationship between TRIM21 and the STING pathway, we hypothesized that TRIM21 interacts closely with components of the STING pathway. First, we evaluated whether TRIM21 binds to STING. In PLA, we observed TRIM21–STING protein interaction from human PBMCs and mouse splenocytes (Supplementary Fig. 4a). In addition, we confirmed that TRIM21 binds directly to STING using IP–western blot assays from human cell line HEK293 (Fig. 5a). Then, considering the known function of TRIM21 as an E3 ubiquitin-protein ligase, we examined whether TRIM21 ubiquitinates STING by overexpressing TRIM21 in the mouse cell line NIH3T3. We observed increased ubiquitination of STING after TRIM21 overexpression (Fig. 5b). Furthermore, we confirmed that TRIM21 functions an E3 ligase that promotes STING degradation through an in vitro ubiquitination assay (Fig. 5c). The structure of TRIM21 includes RING, B-box, coiled-coil and PRYSPRY domains. To determine which domain mediates the interaction with STING, we performed a domain-mapping assay. In the PLA, a significant reduction in PLA-positive cells was observed in NIH3T3 cells overexpressing pmCherry-C1-*Trim21ΔRING-Box* (Fig. 5d). In addition, IP–western blot analysis using NIH3T3 cells overexpressing pmCherry-C1 vector demonstrated that STING ubiquitination was enhanced by *Trim21* WT and *Trim21ΔPRYSPRY*, but not by *Trim21ΔRING-Box* (Fig. 5e). Collectively, these results indicate that the RING-Box domain of TRIM21 is essential for the TRM21–STING interaction. Ubiquitinated proteins can be degraded in either the proteasomes or lysosomes^25^. Using blockers of each pathway (MG132, a proteasome inhibitor; NH_4_Cl, a lysosome inhibitor), we confirmed that degradation of STING after ubiquitination by TRIM21 was recovered only by MG132, indicating that degradation occurs through the proteasome pathway (Fig. 5f). Next, we evaluated whether expression levels of TRIM21 and STING, directly correlate with each other in the lupus-prone mice. In B6.*lpr* mice, the expression of TRIM21 protein decreased whereas that of STING increased compared with B6 mice (Supplementary Fig. 4b). Gene expression levels, analyzed by qPCR, showed the same tendency but no statistical significance was observed in their inverse correlation (Supplementary Fig. 4c). There was no significant difference in the expression levels of IFI16 and DDX41 (Supplementary Fig. 5a). In *Trim21*^−/−^ mice, the protein expression levels of STING were higher than those in WT mice, implying STING pathway activation (Fig. 5g). In the light of results showing higher IFNα-producing pDCs in *Trim21*^−/−^ mice (Fig. 2d), we evaluated whether this is mediated by activated STING pathway. When H-151, a STING antagonist was used, the proportions of IFNα-producing pDCs were decreased under TRIM21-deficient conditions, indicating that TRIM21 deficiency-induced activation of IFNα expression occurs via the STING pathway (Fig. 5h).

### TRIM21 is decreased and correlates negatively with STING in patients with SLE

**Fig. 6:**
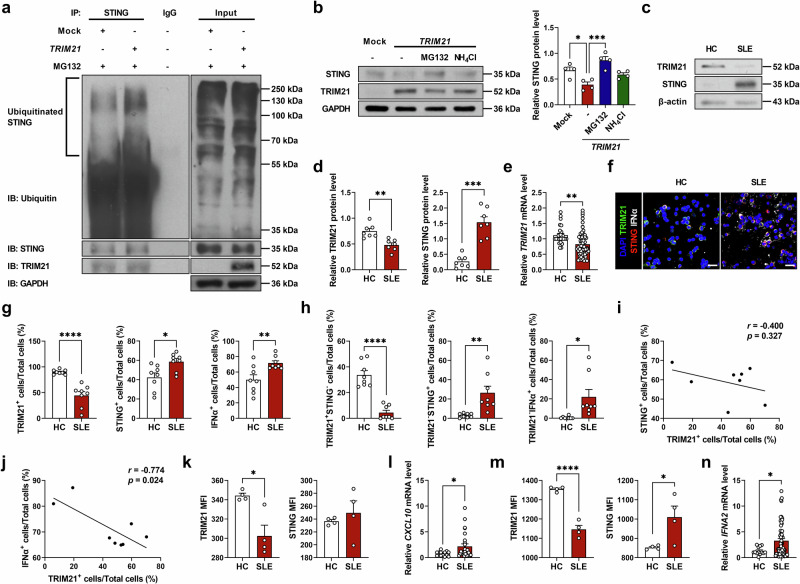
TRIM21 is decreased and correlates negatively with STING in patients with SLE. **a** HEK293 cells were transfected with mock or human *TRIM21* overexpression vector (3 μg) with MG132 (5 μM) treatment. The cells were immunoprecipitated with anti-STING antibodies or normal IgG, and immunoblotted using anti-ubiquitin, anti-STING and anti-TRIM21 antibodies. **b** Western blots and densitometry analysis of the cells for STING and TRIM21. HEK293 cells were treated with MG132 (1 μM) or NH_4_Cl (1 μM). **c**, **d** Western blots (**c**) and densitometry analysis (**d**) of PBMCs from HCs (*n* = 7) and patients with SLE (*n* = 7) for TRIM21 and STING. **e** qPCR of PBMCs from HCs (*n* = 26) and patients with SLE (*n* = 58) for *TRIM21*. **f** Confocal images of PBMCs from HCs (*n* = 8) and patients with SLE (*n* = 8) for TRIM21 (green), STING (red) and IFNα (white) with DAPI (blue) (scale bar, 20 μm). **g**, **h** Stained cell counts of TRIM21^+^ cells, STING^+^ cells, and IFNα^+^ cells (**g**) or TRIM21^+^STING^−^ cells, TRIM21^−^STING^+^ cells, and TRIM21^−^IFNα^+^ cells (**h**). **i**, **j** Correlation between stained cell counts in patients with SLE. Correlation of TRIM21^+^ cell counts and STING^+^ cell counts (**i**) or TRIM21^+^ cell counts and IFNα^+^ cell counts (**j**). **k**, **m** Flow cytometric analysis of TRIM21 and STING in PBMCs from HCs (*n* = 4) and patients with SLE (*n* = 4). The bar graphs represent the MFI values analyzed in CD19^+^ B cells (**k**) and CD11c^+^ DCs (**m**). **l** qPCR of PBMCs from HCs (*n* = 13) and patients with SLE (*n* = 26) for *CXCL10*. **n** qPCR of PBMCs from HCs (*n* = 13) and patients with SLE (*n* = 58) for *IFNA2*. All data are shown as mean ± s.e.m. Statistical analyses were performed using one-way ANOVA (**b**), two-tailed paired *t*-test (**d**, **e**, **g**, **h** and **k**–**n**) or Pearson’s correlation analysis (**i** and **j**). *P < 0.05, **P < 0.01, ***P < 0.001, ****P < 0.0001.

To determine the clinical relevance of our findings, we evaluated the expression levels of TRIM21 and STING in patients with SLE and sought correlations with clinical parameters. First, we determined whether TRIM21 directly binds to and degrades STING through the ubiquitin–proteasome pathway in human cells as it does in murine cells. Using IP–western blot assays and proteasomal or lysosomal inhibitors, we confirmed that, like in murine cells, TRIM21 ubiquitinates STING and degrades it through the proteasome pathway in human cells (Fig. 6a, b). Then, we measured the protein levels of TRIM21 and STING in PBMCs from healthy controls (HCs) and patients with SLE. Western blot assays showed that the expression of TRIM21 protein significantly decreased while that of STING increased in PBMCs from patients with SLE compared with HCs (Fig. 6c, d). In addition, DDX41 showed no difference between the two groups and IFI16 was decreased in patients with SLE compared with HCs (Supplementary Fig. 5b). *TRIM21* mRNA expression levels measured by qPCR in PBMCs were also significantly lower in patients with SLE than in HCs (Fig. 6e). Confocal microscopy of PBMCs showed that TRIM21-stained cells were significantly fewer while STING- or IFNα-stained cells increased in patients with SLE (Fig. 6f, g). In addition, the proportions of cells stained with TRIM21 but not with STING significantly decreased, whereas those of cells stained with STING or IFNα but not with TRIM21 increased in PBMCs of patients with SLE (Fig. 6h), indicating a negative correlation between the levels of expression of TRIM21 and STING (Fig. 6i) or IFNα (Fig. 6j). Flow cytometric analyses of PBMCs revealed significantly reduced TRIM21 and elevated STING in CD19^+^ B cells of patients with SLE (Fig. 6k) with higher *CXCL10* gene expression (Fig. 6l). In CD11c^+^ DCs from patients with SLE, TRIM21 expression was also significantly decreased, whereas STING and *IFNA2* expression was increased (Fig. 6m, n).

### TRIM21/STING imbalance correlates with higher IFN expression and autoantibody production in patients with SLE

**Fig. 7:**
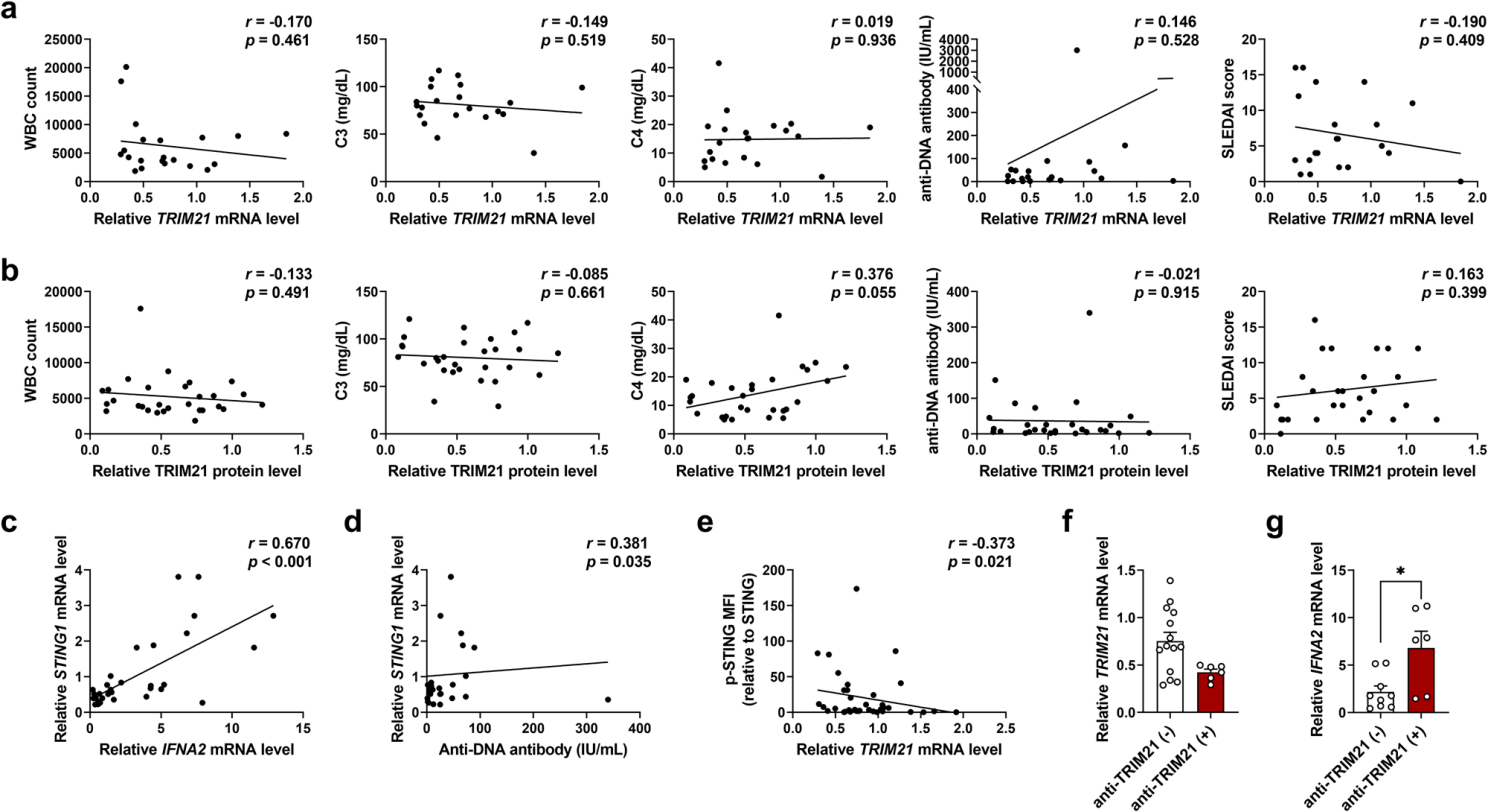
TRIM21/STING imbalance correlates with higher IFN expression and autoantibody levels in patients with SLE. **a**, **b** Correlations between *TRIM21* mRNA (**a**) and TRIM21 protein (**b**) expression levels in PBMCs and disease activity-related clinical parameters, including white blood cell (WBC) counts, complement (C3 and C4) levels, anti-DNA antibody levels and SLE disease activity index (SLEDAI) in patients with SLE (*n* = 21). *TRIM21* mRNA and TRIM21 protein expression were measured by qPCR and western blots, respectively. **c**, **d** Correlations between *STING1* mRNA expression levels in PBMCs and *IFNA2* mRNA expression levels (**c**) and anti-DNA antibody levels (**d**) in patients with SLE (*n* = 31). *STING1* and *IFNA2* mRNA expression were measured by qPCR. **e** Correlations between activated STING (MFI values of p-STING relative to total STING) and *TRIM21* mRNA expression levels in PBMCs of patients with SLE (*n* = 38). The expression of STING and p-STING was measured by flow cytometry. **f**, **g** mRNA expression levels of *TRIM21* (**f**) and *IFNA2* (**g**) in PBMCs from anti-TRIM21 antibody-positive (*n* = 6) and antibody-negative (*n* = 14) patients with SLE measured by qPCR. All data are shown as mean ± s.e.m. Statistical analyses were performed using Pearson’s correlation analysis (**a**–**e**) or two-tailed paired *t*-test (**f** and **g**). *P < 0.05.

Lastly, we evaluated whether the expression levels of TRIM21 or STING in PBMCs correlate with clinical parameters of SLE disease activity. Neither mRNA nor protein expression levels of TRIM21 in PBMCs from patients with SLE correlated directly with systemic disease activity parameters of SLE, including white blood cell counts, complement (C3 and C4) levels, dsDNA antibody levels and SLE disease activity index^26^ (Fig. 7a, b). However, mRNA expression levels of *STING1* in PBMCs of patients with SLE correlated positively with *IFNA2* expression levels (Fig. 7c) and dsDNA antibody levels (Fig. 7d). Expression levels of activated STING, defined as the relative expression of phosphorylated STING (p-STING) to total STING, showed a negative correlation with *TRIM21* mRNA expression in PBMCs from patients with SLE (Fig. 7e). Interestingly, the presence of antibodies against TRIM21 in sera was associated with *TRIM21* and *IFNA2* mRNA expression levels (Fig. 7f, g). Although *TRIM21* showed no statistical significance (Fig. 7f), *IFNA2* mRNA expression levels significantly increased (Fig. 7g) in patients with SLE, suggesting a potential relationship between autoantibody formation and TRIM21 expression levels. Collectively, decreased TRIM21 expression in the PBMCs of patients with SLE correlated with higher STING expression and, thereby, with increased IFN gene expression and dsDNA antibody production.

## Discussion

In the present study, we have shown that TRIM21 regulates the pathological production of type I IFN in murine and human lupus by directly interacting with STING. TRIM21 deficiency induced exaggerated lupus-mimicking phenotypes and increased IFN production along with an activated STING pathway in both induced and spontaneous murine lupus. Furthermore, TRIM21 overexpression attenuates autoimmunity and related inflammation in MRL/*lpr* mice, indicating an important regulatory role in the pathogenesis of SLE. TRIM21 directly interacts with STING and contributes to its degradation through the ubiquitin–proteasome pathway. Lastly, we observed a strong correlation between the expression levels of *STING1* and *IFNA2* in PBMCs from patients with SLE.

Although type I IFNs are essential cytokines in antiviral defense^27^, excessive and prolonged expression of type I IFNs can lead to an aberrant immune response as seen in autoimmune diseases^28^. In previous studies, TRIM21 had been suggested to serve as a negative regulator of IFN production by acting as an ubiquitin ligase, affecting various cellular proteins^29^. Several IFN production-related molecules have been considered to be substrates of TRIM21-mediated ubiquitination. Specifically, IRF3 stimulated by TLR3 activation^30^, and IRF7 following TLR7 or TLR9 stimulation^31^, was degraded through polyubiquitination mediated by TRIM21. Proteins belonging to ‘non-TLR’-mediated pathways have also been reported to be targets of TRIM21. IFI16, an important viral DNA sensor, was reported to be degraded via TRIM21-mediated ubiquitination^20^. DDX41, another cytosolic DNA sensor that stimulates production of type I IFNs, was also reported to be negatively regulated by TRIM21^19^. Both IFI16 and DDX41 use the STING pathway to induce type I IFN production. Although these studies have demonstrated a close relationship between TRIM21 and STING, there is lack of evidence that they interact directly. Previously, it was reported that TRIM21 deficiency in lupus-prone mice advances lupus-related pathology by promoting B cell differentiation and differentiation to plasmablasts^18^. The present study has revealed that TRIM21 can mitigate the STING-mediated production of type I IFN in murine lupus models.

Despite several lines of evidence demonstrating the importance of the STING pathway in inflammatory diseases^13^, its role in lupus pathogenesis has been poorly studied. The levels of cyclic GMP–AMP are elevated in sera from about 15% of patients with SLE, indicating cGAS activation^32^ and possible subsequent activation of STING. However, early studies of the STING pathway using several lupus murine models showed contradictory results. STING deficiency in lupus-prone MRL/*lpr* mice exacerbated lupus-like phenotypes^33^, whereas genetic depletion of STING in *Fcgr2b*-deficient lupus-prone mice protected against disease manifestations, including glomerulonephritis and autoantibody levels^14^. Discrepancies in the role of the STING pathway in lupus-prone mice may reflect the heterogeneity of the involved pathogenic pathways. In addition, considering the therapeutic effects of STING antagonists in lupus-prone mice^34^, it can be posited that activation of the STING pathway can instigate development of SLE. The findings reported herein also support that activation of STING can contribute to the pathogenesis of lupus. Previous studies have shown that TRIM21 interacts with proteins of the STING pathway, including IFI16, DDX41 and IRF3^19,20,30^. Here, we demonstrate a direct binding of TRIM21 to STING leading to its ubiquitination and proteasomal degradation.

Our study has several limitations. First, we have suggested that the main mechanism leading to increased production of IFN in the absence of TRIM21 involves the STING pathway. To fully confirm this concept, it should be shown that pharmacologic inhibition of STING can reverse the effect of TRIM21 deficiency on type I IFN production. Second, due to the relatively small number of patients with SLE included, analyses of the expression levels of TRIM21 and STING stratified by organ involvement, such as skin, kidney or central nervous system, were not performed. This is particularly important considering the previous claims that antibodies against TRIM21 (Ro52) are linked to skin involvement^35,36^. Along these lines, prospective studies are needed to confirm our claims. Third, our study has not addressed the mechanisms that lead to decreased expression of TRIM21 in patients with SLE. Fourth, it is unclear whether antibodies to TRIM21 in patients with SLE are responsible for the decreased TRIM21 levels. We did not directly investigate the role of anti-TRIM21 antibodies in this study. If a direct cause-and-effect relationship is established in future research, blockade of these antibodies may provide clinical benefit in selected patients. Furthermore, our findings suggest that TRIM21 may regulate not only STING protein levels but also STING mRNA expression. However, the mechanism by which TRIM21 influences STING transcription remains unclear. Further studies are needed to determine whether TRIM21 is involved in both posttranslational and transcriptional regulation of STING, including potential effects on gene expression mediated by transcription factors such as IRF3.

In summary, we report that the E3 ubiquitin ligase TRIM21 binds to and degrades STING, thereby regulating type I IFN production in SLE. As blockade of IFN signaling does not provide clinical benefit for all patients with SLE^37^, it is crucial to better understand the underlying molecular pathways responsible for its overproduction. Our findings provide mechanistic insights into the role of the TRIM21–STING pathway, which may be considered as a therapeutic target, whether through direct inhibition of STING, prevention of its proteasomal degradation or restoration of TRIM21 levels.

## Supplementary information

Supplementary Information

## Data Availability

The data that support the findings of this study are available from the corresponding author upon reasonable request.
