# Supplementary material for: The ubiquitin E3 ligase TRIM21 suppresses type I interferon signaling via STING degradation and ameliorates systemic autoimmunity
Source: Exp Mol Med. 2025 Jul 3;57(7):1555–66. doi: 10.1038/s12276-025-01490-5 (PMC12322227; doi:10.1038/s12276-025-01490-5)
Supplement: Supplementary file 1 — Supplementary Information [file 12276_2025_1490_MOESM1_ESM.pdf]

Supplementary Fig. 1: The STING pathway is activated and TRIM21 is reduced in B cells and pDCs of MRL/*lpr* mice.

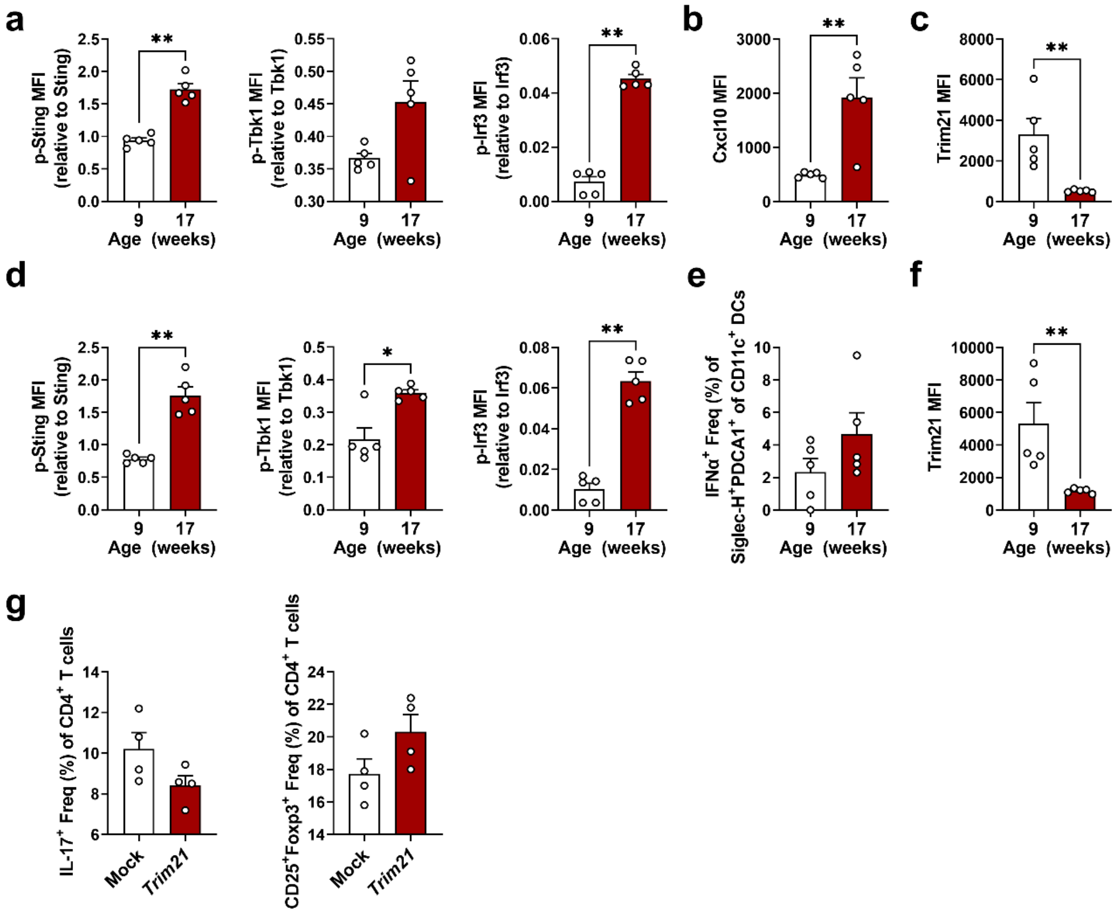

a–f Flow cytometric analysis in splenocytes from female MRL/*lpr* mice (9 weeks, n = 5; 17 weeks, n = 5). The STING pathway-related proteins (**a**, **b**, **d**, **e**) or Trim21 (**c**, **f**) were analyzed in CD19^{+} B cells (**a–c**) or Siglec-H^{+}PDCA1^{+} Freq (%) of CD11c^{+} DCs (**d–f**). Values are MFI (**a–d**, **f**) or percentages of positive cells (**e**). **g** Eight-week-old female MRL/*lpr* mice were injected weekly with mock (n = 4) or mouse *Trim21* overexpression (n = 4) vector for 8 weeks. Flow cytometric analyses of Th17 and Treg cells in splenocytes of 16-week-old MRL/*lpr* mice. Values are percentages of positive cells. All data are shown as mean ± SEM. Statistical analyses

were performed by two-tailed paired *t*-test. $*p < 0.05$, $**p < 0.01$.

Supplementary Fig. 2: Inflammatory B cells are expanded under TRIM21-deficiency conditions, and inflammatory T cells and IFNα are increased in *Trim21*$^{-/-}$ mice following treatment with R848.

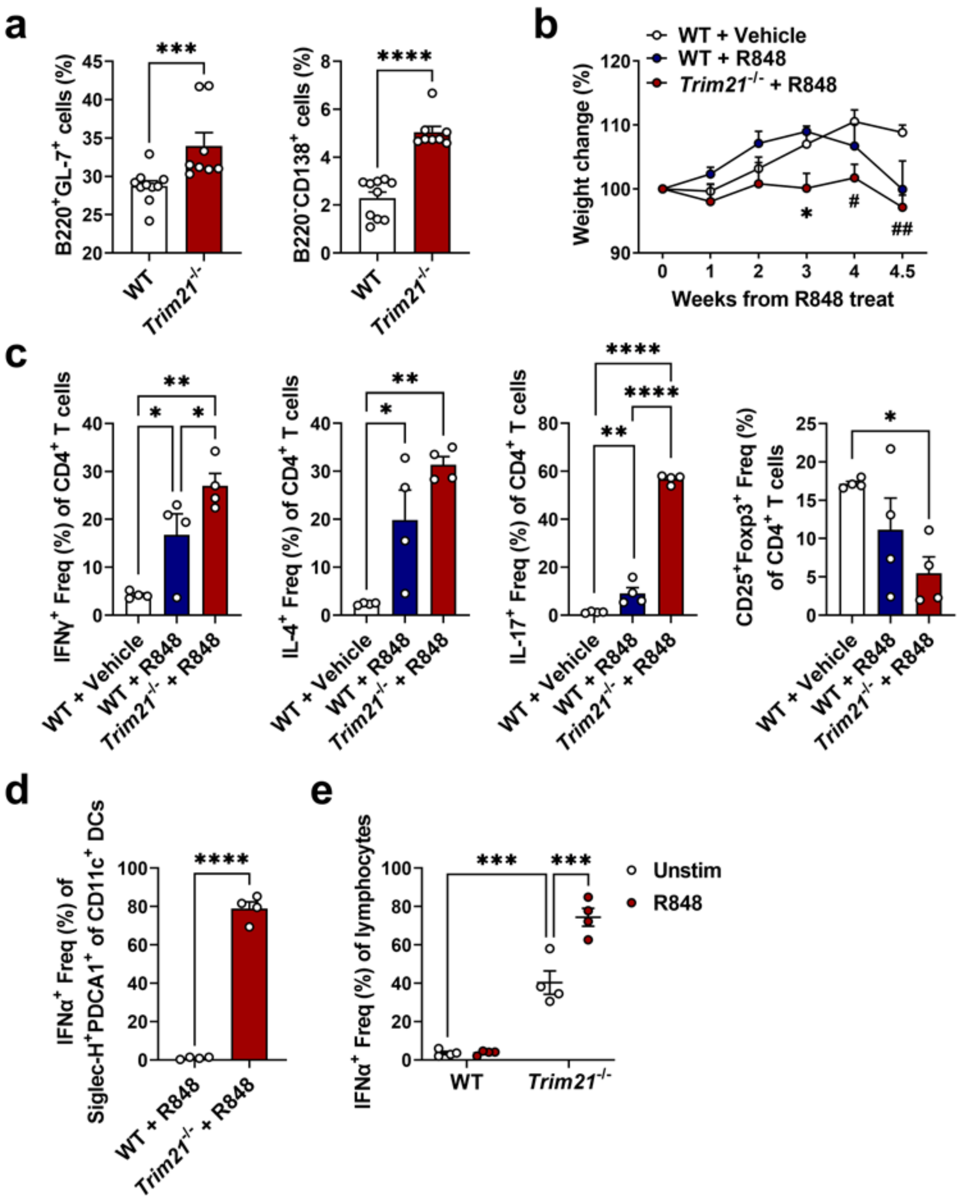

a Splenic B220^{+} B cells of WT B6 and *Trim21*$^{-/-}$ mice were stimulated with CD40 Ligand, anti-IgM, and IL-4 for 3 days. GC B cells and plasma B cells were analyzed by flow cytometry. **b**–

d Eight-week-old female WT B6 and *Trim21*$^{-/-}$ mice were treated with vehicle (acetone alone, n = 4) or R848 (n = 4) for 31 days. **b** Changes in body weight of the mice (*compared with WT + R848 group, #compared with WT + Vehicle group). **c**–**d** Flow cytometric analysis of Th1, Th2, Th17, and Treg cells (**c**) and IFNα-producing pDCs (**d**) in splenocytes. Values are percentages of positive cells. **e** Splenocytes of WT B6 or *Trim21*$^{-/-}$ mice were stimulated with 1 μg/ml R848 for 2 days. IFNα-producing lymphocytes were analyzed using flow cytometry. Values are percentages of positive cells. All data are shown as mean ± SEM. Statistical analyses were performed by two-tailed paired *t*-test (**a**, **d**), two-way ANOVA (**b**, **e**), or one-way ANOVA (**c**). *$p < 0.05$, **$p < 0.01$, ***$p < 0.001$, ****$p < 0.0001$. #$p < 0.05$, ##$p < 0.01$.

Supplementary Fig. 3: TRIM21 deficiency exacerbates lupus manifestations in B6.*lpr* mice.

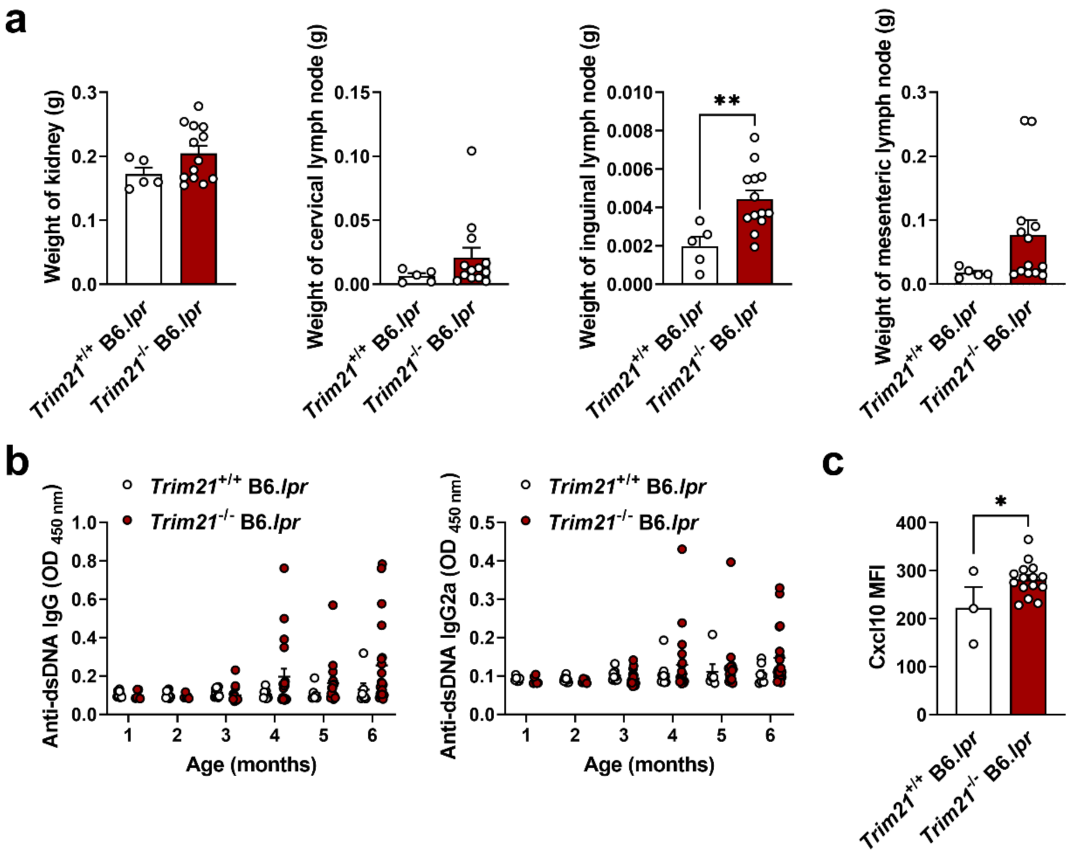

a Kidney, cervical lymph node, inguinal lymph node, and mesenteric lymph node weights of 7-month-old *Trim21*$^{+/+}$ (n = 5) and *Trim21*$^{-/-}$ (n = 13) B6.*lpr* mice. **b** Levels of anti-dsDNA antibodies (total IgG and IgG2a) were measured from serum of *Trim21*$^{+/+}$ and *Trim21*$^{-/-}$ B6.*lpr* mice using ELISA. **c** Flow cytometric analysis of Cxcl10 in splenocytes of 7-month-old *Trim21*$^{+/+}$ (n = 3) and *Trim21*$^{-/-}$ (n = 15) B6.*lpr* mice. Data were analyzed in CD19^{+} B cells and shown as MFI values. All data are shown as mean ± SEM. Statistical analyses were performed by two-tailed paired *t*-test (**a**, **c**) or two-way ANOVA (**b**). *$p < 0.05$, **$p < 0.01$.

Supplementary Fig. 4: TRIM21 interacts with STING.

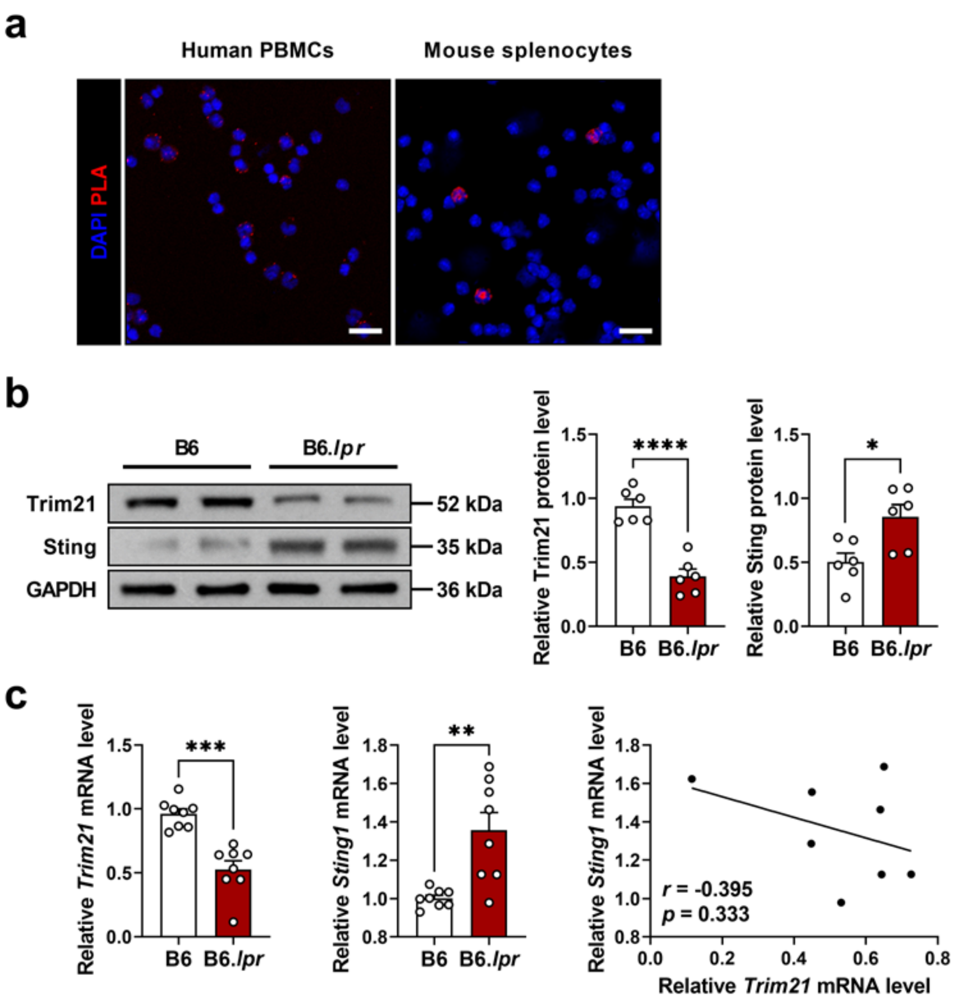

a PLA to detect protein interactions between TRIM21 and STING in human PBMCs and mouse splenocytes. Human PBMCs were obtained from healthy donors, and mouse splenocytes were obtained from WT B6 mice. Each red spot represents for a single interaction and nuclei was stained with DAPI (scale bar: 20 µm). **b** Western blots and densitometry analysis of splenocytes from female B6 and B6.*lpr* mice for Trim21 and Sting. **c** mRNA levels of *Trim21* and *Sting1* in splenocytes from B6 (n = 8) and B6.*lpr* (n = 8) mice analyzed by qPCR. Correlations of these genes in B6.*lpr* mice are shown in the right panels. All data are shown as mean ± SEM.

Statistical analyses were performed by two-tailed paired *t*-test (**b**, left and middle panels in **c**) or Pearson's correlation analysis (right panels in **c**). *$p < 0.05$, **$p < 0.01$, ***$p < 0.001$, ****$p < 0.0001$.

Supplementary Fig. 5: IFI16 and DDX41 are not targets of TRIM21.

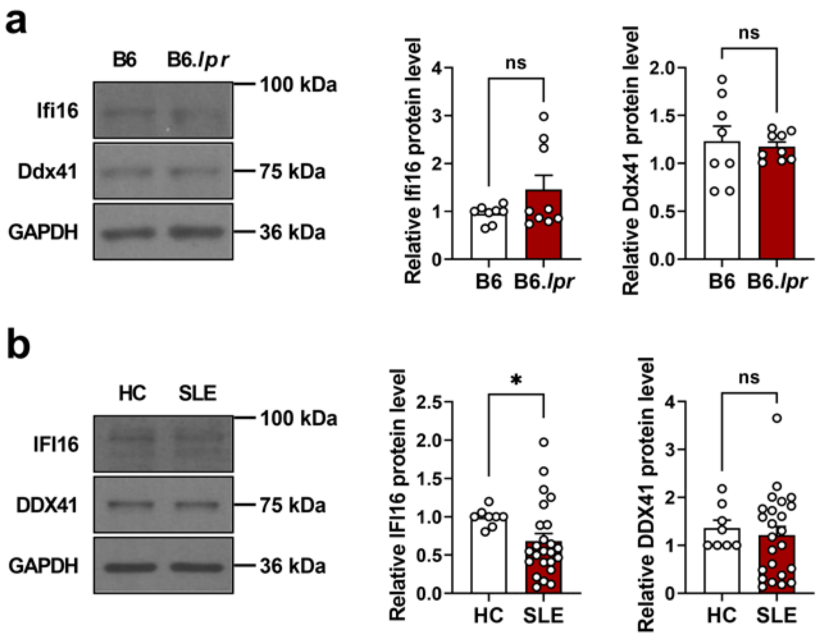

a Western blots and densitometry analysis in splenocytes from female B6 (n = 8) and B6.*lpr* (n = 9) mice for Ifi16 and Ddx41. **b** Western blots and densitometry analysis in PBMCs from HC (n = 8) and SLE patients (n = 24) for IFI16 and DDX41. All data are shown as mean ± SEM. Statistical analyses were performed by two-tailed paired *t*-test. $*p < 0.05$.
